# Supplementary material for: Prediction of gastrointestinal functional state based on myoelectric recordings utilizing a deep neural network architecture
Source: PLoS One. 2023 Jul 27;18(7):e0289076. doi: 10.1371/journal.pone.0289076 (PMC10374095; doi:10.1371/journal.pone.0289076)
Supplement: S1 Table — In the table, a heat map is used to highlight performance (red = high percentage to green = low percentage). Results were obtained with a detection threshold of 0.5. We show the average and standard deviation of the forward and backward methods (8 methods for each) to summarize them. (DOCX) [file pone.0289076.s001.docx]

**Supplemental Table 1: Performance of classifiers per animal**

|  |  | **103-21** | |  | **58-21** | |  | **60-21** | |  | **87-21** | |
| --- | --- | --- | --- | --- | --- | --- | --- | --- | --- | --- | --- | --- |
|  |  | Average | SD |  | Average | SD |  | Average | SD |  | Average | SD |
| Forward Selection Methods | **Accuracy %** | 61.5 | 10.8 |  | 73.1 | 8.6 |  | 74.8 | 12.1 |  | 73.9 | 6.3 |
|  | **Sensitivity %** | 55.2 | 13.3 |  | 67.5 | 12.4 |  | 69.4 | 17.8 |  | 77.7 | 7.0 |
|  | **Specificity %** | 67.7 | 12.2 |  | 78.8 | 7.2 |  | 80.2 | 9.0 |  | 70.0 | 6.1 |
|  |  |  |  |  |  |  |  |  |  |  |  |  |
| Backward Selection Methods |  | Average | SD |  | Average | SD |  | Average | SD |  | Average | SD |
|  | **Accuracy %** | 69.9 | 3.0 |  | 77.7 | 3.0 |  | 79.0 | 5.9 |  | 75.1 | 4.3 |
|  | **Sensitivity %** | 65.4 | 8.7 |  | 75.4 | 6.9 |  | 73.5 | 11.5 |  | 80.2 | 5.2 |
|  | **Specificity %** | 74.4 | 5.5 |  | 80.0 | 4.0 |  | 84.4 | 4.0 |  | 70.0 | 7.1 |
|  |  |  |  |  |  |  |  |  |  |  |  |  |
| CNN | **Accuracy %** | 90.0 |  |  | 92.5 |  |  | 91.7 |  |  | 85.0 |  |
|  | **Sensitivity %** | 91.7 |  |  | 93.3 |  |  | 90.0 |  |  | 78.3 |  |
|  | **Specificity %** | 88.3 |  |  | 91.7 |  |  | 93.3 |  |  | 91.7 |  |

In the table, a heat map is used to highlight performance (red = high percentage to green = low percentage). Results were obtained with a detection threshold of 0.5. We show the average and standard deviation of the forward and backward methods (8 methods for each) to summarize them.
